# Dynamic Voltage-Frequency and Workload Joint Scaling Power Management for Energy Harvesting Multi-Core WSN Node SoC

**DOI:** 10.3390/s17020310

**Published:** 2017-02-08

**Authors:** Xiangyu Li, Nijie Xie, Xinyue Tian

**Affiliations:** Tsinghua National Laboratory for Information Science and Technology, Institute of Microelectronics, Tsinghua University, Beijing 100084, China; xnj14@mails.tsinghua.edu.cn (N.X.); txy12@mails.tsinghua.edu.cn (X.T.)

**Keywords:** task scheduling, power management, energy harvesting, WSN

## Abstract

This paper proposes a scheduling and power management solution for energy harvesting heterogeneous multi-core WSN node SoC such that the system continues to operate perennially and uses the harvested energy efficiently. The solution consists of a heterogeneous multi-core system oriented task scheduling algorithm and a low-complexity dynamic workload scaling and configuration optimization algorithm suitable for light-weight platforms. Moreover, considering the power consumption of most WSN applications have the characteristic of data dependent behavior, we introduce branches handling mechanism into the solution as well. The experimental result shows that the proposed algorithm can operate in real-time on a lightweight embedded processor (MSP430), and that it can make a system do more valuable works and make more than 99.9% use of the power budget.

## 1. Introduction

Energy harvesting wireless sensor network (WSN) nodes which can harvest energy from the environment during operation have gained a lot of interest recently [1,2,3,4]. Under this situation, the goal of power management is no longer to minimize the power consumption but to acclimatize the operations to varying power so as to make sustainable and full use of harvested energy. Meanwhile, the expansion of applications requires greater processing ability of WSN nodes, and hence results in the potential utilization of the heterogeneous multi-core SoC in WSN nodes, in which processing elements (PE) can be general-purpose processors, specialized hardware accelerators, application specified processors or reconfigurable elements, as proposed in [5,6]. This kind of system usually adopts dynamic voltage-frequency scaling (DVFS) and some other low power techniques to improve its energy efficiency, thus has multiple power states. The traditional power management strategy for single core and battery-powered system is not suitable for these kind of system, a new method including task scheduling and power management is required. Additionally, WSN applications have some special characteristics. First, WSN nodes are resource-limited systems, so in such a situation the full utilization of all available power to improve an aspect of performance is very important. Second, the application of WSNs is modular, and there are usually multiple stand-alone and concurrent programs in the system. Third, the power consumption of most the WSN applications’ programs are data dependent. The power management solution should also consider these characteristics. This paper addresses the problems of scheduling and power management of hard deadline tasks in a light-weight energy harvesting heterogeneous multi-core WSN node system, which is a real-time embedded system that supports PE-granularity DVFS technique.

There are some special considerations in this problem: at first, a more complicated model of the goal and constraints of power management should be considered. First, for an energy harvesting system, the energy is relatively infinite but the power budget is constrained. Under the prerequisite that the node is kept working at a requested performance level without power failure as long as possible, the temporary excess harvested energy can be better utilized to improve the service quality of the system, i.e., to do more valuable works, because the energy storage always has size limitation and energy loss. Several possible different aspects to be improved can be selected, such as sampling rate, accuracy, reliability, etc., depending on the application. These aspects of performance are often sacrificed for limitation on power. Therefore, there are sizable potential enhancing spaces in these aspects when the system is permitted to increase workload, such as the amount of operations and transmissions. The workload scalability can provide an alternative approach for power tuning. The combination of voltage/frequency adjustment and workload scaling provides the designer a larger design space and also makes the problem more challenging. Second, a challenge for the power management of a multi-task and multi-core system is that the power budget is not only programmed over different time intervals, but also allocated to different heterogeneous cores and tasks. The power management problem includes not only determination of power budget for every time instant but also the utilization of these budgets. The determination of power budget can use the methods proposed for single processor systems for reference, which are widely studied, hence this paper mainly focuses on the method of obtaining the best energy utilization efficiency for multi-core systems under a given power budget. For a heterogeneous multi-core SoC, this involves mapping from tasks to PEs and allocation of power budget among them, which is much more complicated compared with the single core scenario. This is an NP-complete problem as is known [7]. Third, the power budget of a time interval is time-varying and hard to predict exactly or control. Therefore, the power management must be performed in real-time. However, the WSN node platforms are resource-constrained therefore the real-time algorithms should have a low complexity.

In this paper, an energy utilization efficiency optimization solution including task scheduling and power management, applied to energy harvesting multi-core WSN node processors is proposed. It has a two-phase framework which integrates a static task scheduling and a dynamic work mode configuration optimization. The proposed method combines both DVFS and workload scheduling. It not only can make full use of power budget to improve the system performance, but also has a simple real-time algorithm so as to run on WSN platforms, which have limited resources. The workload is used as a unified intermediary for different aspects of performance. And a compositive metric figure, system reward is defined for unified measurement of improvement of performance and as the optimization goal. So this method has relatively high versatility.

This paper is organized as follows: Section 2 reviews previous related works. Section 3 describes the problem and some basic concepts. Section 4 introduces a task scheduling algorithm. In Section 5 a greedy configuration optimization is presented, and the result is examined in Section 6.

## 2. Related Works

Early studies on power management mainly focus on minimizing the power consumptions under given performance requirements [8] or maximizing the performance under a given power budget using DVFS or other dynamic power management techniques [4]. The constraints in aspect of power may be the total energy of a battery powered system, or the power budget coming from temperature limitation, for example.

However for the energy harvesting systems, the constraints are quite different. Unlike the battery powered systems, their energy supply is relative infinite but the power consumption is limited. One major concern for an energy harvesting node is to ensure the node remains alive as long as possible [2]. The optimization goals under this constraint are more diverse. Kansal et al. have proposed the concept of energy-neutral to determine power budget, aiming at keeping the node work on a desired performance level forever and have suggested a method to dynamically adjust duty cycle of the processor to maximize the utility [9]. Other metrics can also be used as the optimization goal. A control scheme combining data and power management has been used in [1], the purpose is to minimize the size of the energy storage device. Many studies have focused on maximizing the energy efficiency or system performance under a given energy budget. The concept of quality of service (QoS), which is defined in terms of delay, throughput, and packet loss is introduced as the goal of optimization in [10]. In [11], a computation system consisting of multiple parallel clock-gated processing unit is introduced. The number of active units is determined dynamically according to the power budget to maximize the system computation speed while not exceeding the available power. The deadline miss rate (DMR) is also widely used as a measure of efficiency of real-time systems. In [12], a modified earliest deadline first algorithm which takes the energy constraint into account has been introduced. The DMR and energy violation rate are used as the measures of the system performance. The system utilization and energy state is jointly considered in [13] to select an operating frequency, aiming at minimizing the DMR. However, the goal of these works can only address the application requirement in one aspect. Hence, the applicability of their methods is limited. In addition, they all assume that all the tasks are of the same importance, however, the importance of different tasks may different in some applications.

Another issue of an energy harvesting system is that the power constraint usually varies with time and the system must be adjusted accordingly in real-time. Therefore, it is necessary for a power management of WSN nodes to have a low cost overhead and can operate in real-time [14]. A task scheduling method considering power switching overhead is introduced in [15], a task splitting strategy is applied to improve the performance and a heuristic strategy is implemented to reduce the problem complexity. One approach to reducing the complexity is the tradeoff between performance and computational cost. A model based on Markov Decision Process has been studied in [16], and a greedy policy is used to reduce the complexity of the algorithm. In [17], a lazy scheduling algorithm focusing on degrade the overhead through tolerating a certain percentage of tasks miss has been proposed. Another commonly used technique is to split the problem into static and dynamic two parts. With this, the computation of the static part can be offloaded from the computation which is executed online. In work [18], a deadline-aware scheduling algorithm with energy migration strategies for the distributed super capacitors has been proposed. The optimal capacitor sizes and Artificial Neural Network training samples are determined offline, and an online part determines the real-time optimal capacitor size, scheduling pattern and task queue dynamically. 

Multi-core SoC architectures have been proposed to be employed in WSN nodes [5,6]. For example, Hempstead et al. proposed an energy-efficient WSN node architecture that fully embraces the accelerator-based computing paradigm, including accelerators for routing and data filtering with accelerator-level VDD-gating. Their system contains five cores besides an event processor and a memory unit [6]. In a multi-core system, especially a heterogeneous system, the power consumption is related to the mapping of tasks to processing elements (PEs) [19,20]. Both the task scheduling and the power state of the PE running every task should be optimized during an energy-harvested-aware system management [21]. This raises new challenge to the power management algorithms. In [22], a method which combines DVFS and an algorithm which allocates the periodical tasks to the core with the lowest utilization is proposed. An algorithm inspired by auction theory has been proposed in [23], the cores bid for the opportunity to be active and their power and clock frequency are gated or scaled according to the power allocation decision.

Voltage and frequency adjustment are the most commonly used methods in power management [24,25] of general purpose systems. However, in some energy-harvesting systems, some tasks may have multiple work modes. Their workloads, which are the amount of operations or transmissions, can be also scaled to result in different power consumption levels. This characteristic provides another approach for power management and can further improve the energy utilization efficiency. For example, a framework exploiting the application’s tolerance to quality degradation is proposed in [26]. It adjusts the quality of collecting data according to the energy harvesting conditions. In addition, an algorithm adapting the sampling frequency according to the available energy has been introduced [27]. The combination of the voltage/frequency adjustment and the workload scaling can achieve a larger optimization space. A joint scaling method is proposed in [28], the method scales the CPU frequency and system voltage while adjusting the radio modulation levels, so as to satisfy the performance requirement and achieve the goal of maximizing the minimum energy reserve. However, the system level solution combining both the DVFS and the workload scaling for heterogeneous multi-core WSN nodes as we proposed has not been studied yet.

## 3. Problem Modeling and Terms

### 3.1. Hardware Description

According to the features of accelerator-based WSN node SoC, the hardware is regarded as a platform consisting of multiple heterogeneous processing elements. A PE can be a general-purpose processor, a specialized hardware accelerator, an application specified processor or a reconfigurable element. The execution times of a type of tasks running on different kinds of PEs are different. Those specialized PEs such as the accelerators can only execute their specified kinds of tasks. First, approximately assume that the supply voltage of each PE can be adjusted separately and continuously within a certain range while the frequency is adjusted accordingly, resulting in different performance and power consumption. So far, the scaling steps of the voltage regulators can be low to several millivolts, therefore this approximation is reasonable. However, the supply voltage remains unchanged during the execution of each task for simplicity. Second, consider the situation that the node is equipped with an energy harvesting system and an energy storage, such as an ultracapacitor or a battery. And there is a power aware structure in the system monitoring the power harvested, the energy stored in the energy storage device, and the power state of each PE. Last, assume that the variation of the power harvested is slow enough to take the power budget as a constant during a scheduling time window.

### 3.2. Application Model

- Program
  A program is the implementation of a function or application of the system. The programs are independently and can run concurrently. For example, a WSN node may run sampling and processing programs for several sensors, a routing program, a location program, and so on separately. A program consists of a group of conjoint tasks executing on various PEs and can be expressed by a Task Flow Graph (TFG). We assume that every program has a hard deadline, considering the WSNs are usually real-time systems. A program will be cancelled when it has not been completed by its deadline. Besides, assume that all programs are periodic as is typical in WSN applications.
- Task and Workload
  Each task has a workload and different executing speeds on different PEs. Besides, some tasks in some programs have multiple work modes, corresponding to different levels of working effort, such as the rate of sensor sampling or self-testing, the data accuracy of processing, or the number of iterations of a learning algorithm. Workload is introduced as a unified measurement of the quantity of the works done by a task. It is the normalized amount of operations of a task. For different tasks of different applications workload can refer to the efforts in different metrics, e.g., the accuracy or reliability. Some tasks have fixed workload, and some tasks have scalable workload. There is a valid range of workload of each task, the lower bound is the least work to be done to maintain the task’s function and the upper bound is the maximum amount of meaningful work within a time window. Excess workload has no contribution to the quality of service.

For a PE, the energy consumption is proportional to the workload and has a corresponding value for each kind of task. Based on the above definitions, the energy consumption of PE_*u*_ executing task_*j*_ under a certain work mode can be expressed as:

(1) $$E_{uj}=Q_{j}C_{uj}{V_{u}}^{2}$$

where *C_uj_* is the equivalent capacitance when executing task_*j*_, which reflects the energy efficiency of a task on the PE, *Q_j_* is the normalized workload of task_j_ and *V_u_* is the supply voltage of the PE_*u*_. The static power consumption is ignored for simplicity.

The difference of execution time of a task executing on different PEs is expressed by a factor, *k*. A task executing on the specific PEs for it such as accelerators and specific processors is faster thus with a smaller *k*. For a general purpose processor, there is usually a penalty in execution time and the value of *k* factor is higher. If task_j_ cannot run on PE_u_, *k_uj_* = ∞. Assuming execution time of a task is proportional to its workload with a coefficient *a_j_*, we write the execution time of task_j_ executing on PE_u_ as:

(2) $$T_{uj}=\frac{a_{j}k_{uj}Q_{j}V_{u}}{{\left(V_{u}-V_{th}\right)}^{2}}$$

System reward is used as a unified measurement of the improvement of quality of service, which represents different metrics such as the computation accuracy, reliability, and the sampling rate of a sensor, comprehensively. The system reward of a program *Pr_i_* is the weighted sum of the rewards of its tasks:

(3) $$U_{i}=\sum w_{j}U_{j},\forall task_{j}\in Pr_{i}$$

For most tasks, improvement in the quality of service involves increasing in workload. It’s natural to assume that system reward of a task is proportional to its workload within a valid range:

(4) $$U_{j}=\{\;\begin{array}{c} 0,\;when\;Q_{j}<Q_{min}\\ l_{j}Q_{j},\;when\;Q_{min}\leq Q_{j}\leq Q_{max}\\ U_{max,j},\;when\;Q>Q_{max} \end{array}$$

### 3.3. Problem Description

The scheduling time window is the hyperperiod of all programs, of which the length is the least common multiple of all the programs’ periods [29]. The sets of programs to be run in every scheduling time window are the same. A scheduling window is composed of a finite number of time slices. A time slice is the minimum basic time unit in scheduling, which can be the minimum of the shortest clock cycles of all PEs. Assume that there is a constraint for the total energy consumption of each time window, which is determined by the energy-harvested-aware management mechanism, and that its corresponding average power over the time window is the power budget. For a certain set of programs, the problem to be solved is to determine the actual start time, workload and PE for each task in addition to its work mode, which corresponds to the supply voltage as well as the clock period of the assigned PE during execution.

The goal of our energy management is to improve the energy utilization efficiency of the system. It aims at maximizing the system reward under a given power budget. As for a power-aware system, the power budget is determined in real-time, it is natural to deploy the task scheduling and power management algorithm on WSN platforms. However in a heterogeneous multi-core SoC system, the scheduling problem is an NP-complete problem with a huge solution space [7], the complexity of the classical solving algorithms are unbearable for the WSN nodes where both energy and performance are limited, so the problem is decomposed into two stages: task scheduling and configuration optimization to lower the complexity. During the task scheduling stage, the power constraint is not taken into account. Hence, it can be done during the design phase, allowing the algorithm to have a higher complexity. The configuration optimization algorithm is in charge of optimizing the configuration for the tasks’ work mode, including the workload and the assigned PE’s supply voltage/frequency of the tasks, based on the result of task scheduling according to the real-time power budget. It is implemented on the WSN platform and therefore should have a low complexity. This 2-phase approach is widely adopted and cuts down the complexity significantly while only causing a little performance degradation [30].

#### 3.3.1. Task Scheduling

The task scheduling problem can be described as an assignment of each task to a certain PE under the timing constraints for a number of input TFGs altogether. Given *N*, the number of PE, and a set of programs, *A*, that contains *L programs* (*Pr*_1_, *Pr*_2_, …, *Pr_L_*) *and M* tasks in total, the problem can be described as follows:

Find the optimal *x_uj_* and *D_j_* for and *j* = 1, …, *M*, so that:

(5) $$\overset{N}{\underset{u=1}{\sum }}\overset{M}{\underset{j=1}{\sum }}x_{uj}=1,\;\mathrm{where}\;x_{uj}\in \left\{0,1\right\}\;for\;u=1,\;\ldots ,\;N\;\mathrm{and}\;j=1,\ldots ,\;M$$

while:

(6) $$D_{j}+x_{uj}T_{uj}\leq deadline_{i},\;\forall task_{j}\in Pr_{i},\;\forall Pr_{i}\in A$$

(7) $$D_{n}-D_{m}-T_{m}\geq 0,\;\forall task_{m}\in precedence\;of\;task_{n}$$

where *x_uj_* represents whether task_*j*_ is assigned to PE_*u*_, *D_j_* is the start time of the task_*j*_, and *T_uj_* is the execution time of task_*j*_ on PE_*u*_. Equations (6) and (7) is the timing constraints. For the hard deadline system we are considering, the timing constraint that tasks must finish executing before deadline must not be violated. The assignment result of the scheduling is the input of the configuration optimization algorithm, and the assignment and execution orders of tasks will remain unchanged during the configuration optimization stage. In addition, for the programs with conditional branching, the impacts of the conditional branching must be taken into account because the lengths of paths of different branches of a WSN program may be quite different. For example, it is a common case that a sensor data processing program has a data dependent branch that if the data is larger than a threshold, a series of processing will be done, else it is ignored without any more operations.

The inputs of the task scheduling are several TFGs, each represents a program to be scheduled. The nodes of the graphs are the tasks of the programs, and each edge *e*(*i*,*j*) represents the dependency between task_*i*_ and task_*j*_, this may be a data dependency or a control dependency. The result of the task scheduling is also represented by a TFG, the nodes of which include the nodes of all input TFGs. The edges of the graph include all the edges inherited from the input graphs and the new edges added due to the hardware resource sharing in scheduling result. If task_*i*_ and task_*j*_ are assigned to the same PE and task_*j*_ is the closest task behind task_*i*_, there will be an edge from task_*i*_ to task_*j*_. The result TFG generated off-line is stored in the system. Its configuration scheme is set as the initial configurations of the tasks.

#### 3.3.2. Configuration Optimization

During the work phase, the configuration optimization procedure is run on the WSN node at the beginning of each time window. At first, the power budget of the coming time window is set by the system. The energy budget is determined considering the energy level of the energy storage device and the harvested energy predicted in this window. The detail determining algorithm has been well studied and is beyond the topic of this paper. And the configuration scheme of the tasks and PEs are then updated by the optimization algorithm according to the power budget in order to obtain a better energy utilization efficiency. For a platform equipped with energy harvesting and storage device, it is acceptable to occasionally violate the power budget in trade for meeting the hard task deadline if must. The system can fill the power gap by the battery storage, and compensate the energy deficit by adopting a more conservative power utilization policy and amortizing the energy deficit in the several incoming time windows, so the power budget is a soft constraint.

The basic principle of the algorithm is to increase the workload of the tasks and scale the supply voltage along with the frequency of the PEs to improve the system rewards while do not violate the power budget and timing deadline constraints. Finally, every task will adopt its configuration stored in the optimized scheme when it really starts executing.

As mentioned above, the result of the task scheduling is a TFG stored in the system. Each path in this TFG has a deadline, which is the deadline of the program to which the end node of the path belongs. The timing slack of a path is defined as the difference between the deadline and the total execution time of it, which is expressed as (8):

(8) $$slack_{k}=deadline_{k}-\underset{task_{j}\in path_{k}}{\sum }T_{uj}$$

Our purpose is to maximize the system rewards under the power budget and timing constraints. The problem for an input TFG, denoted by *G*(*t*,*e*), where ***t*** is the set of the nodes of *G* and *e* is the set of its edges, can be expressed as an optimization problem of determining the *V_uj_* and *Q_j_* for *u* = 1,…,*N* and *j* = 1,…,*M* so that:

(9) $$\max:\overset{L}{\underset{i=1}{\sum }}\sigma_{i}U_{i}$$

while:

(10) $$\overset{N}{\underset{u=1}{\sum }}\overset{M}{\underset{j=1}{\sum }}x_{uj}E_{uj}\leq E_{B}$$

(11) $$slack_{k}\geq 0,\forall path_{k}\;\mathrm{of}\;G$$

(12) $$Q_{min,j}\leq Q_{j}\leq Q_{max,j},\;\forall task_{j}\in t$$

(13) $$V_{min}\leq V_{uj}\leq V_{max},\;\forall PE_{u}\;\mathrm{and}\;\forall task_{j}\in t$$

(14) $$D_{n}-D_{m}-T_{m}\geq 0,\;\forall task_{m}\in t,\;task_{n}\in t\;\mathrm{and}\;\exists e\left(m,n\right)\in e$$

where *E_uj_* is the energy consumption of task_*j*_ executing on PE_*u*_, the total energy consumption should not exceed the power budget *E_B_*. is the weighting factor of the reward of *Pr_i_* belonging to the application ***A***. Task workload *Q_j_* and PE supply voltage *V_uj_* is scaled continuously within the certain ranges to maximize the total system reward. It is a non-linear programming problem.

## 4. Task Scheduling Algorithm

During the task scheduling stage, the tasks are assigned to PEs and their starting times are determined. In order to obtain a greater potential for improving workload in the configuration optimization stage, the tasks are set to their minimum workload and the PEs are configured to the lowest supply voltage in the scheduling stage. A priority-based task scheduling algorithm aimed at maximizing the path delay slacks based on the algorithm proposed in [31] is applied. The goal of this algorithm is to put the unrelated tasks on different paths and let less tasks constrained by tighter deadlines, so that we can derive looser constraints and a larger optimal space. Each task is assigned a priority, which is defined as the sum of the latest finishing time and the earliest starting time. And then the tasks are assigned to the PEs in order of their priorities. If several PEs are available when a task is ready, the PE with the latest available time will be chosen, or else the first PE available after the ready time of the task will be chosen.

The algorithm of [31] is modified to suit the accelerator equipped scenario. When assign tasks, the PEs are sorted by *k_uj_*, the PE with a smaller *k* is chosen first. Thus, it tends to assign a task to the specialized PE for it. The general purpose processor will be considered only when the specialized processors are not available.

**Figure 1:**
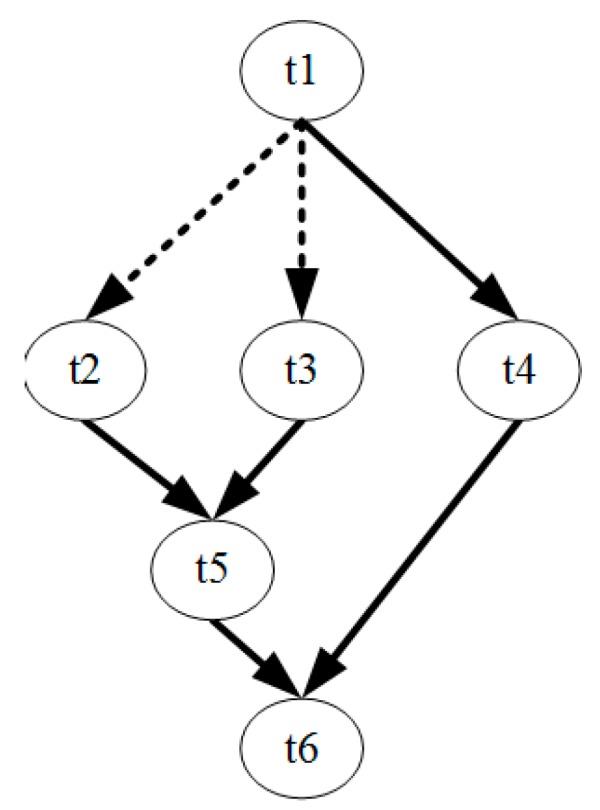
A conditional branching TFG.

Regarding the TFG containing conditional branches, whether a task is executed depends on the result of the preceding tasks. For example, in the task flow shown in Figure 1, where the dash lines represent conditional branches, task t2 and task t3 is expected to be executed after t1 but not both. The algorithm of [31] cannot handle this kind of case. Consequently, we introduce a mutual exclusive mechanism into it.

Two tasks are regarded as mutual exclusive if they locate on different branch paths that have conflicting branch conditions. To identify the mutual exclusive tasks, a branch labeling method proposed in [32] is used. Each task is associated with a branch level, which is the depth of the current branch. In addition, for each branch executed before reaching this task, a branch label is added to the task, which indicates the execution conditions of this task at that branch. The rule of examining whether two tasks are mutual exclusive is as follows:

If the branch level is zero, the tasks are not exclusive. Else, if the minimum of the depths of the two tasks is *d*, then compare the first *d* labels of these two tasks in the order of the branch depth. If all *d* labels are not equal, these two tasks are exclusive.

If two tasks are mutual exclusive, no more than one of them will be executed at the same time under a certain circumstance. During the task scheduling, the mutual exclusive tasks can share the same PE and can have overlapping execution time. Using this mechanism, the scheduling algorithm is modified accordingly in addition to have the branch handling ability.

## 5. Configuration Optimization

**Figure 2:**
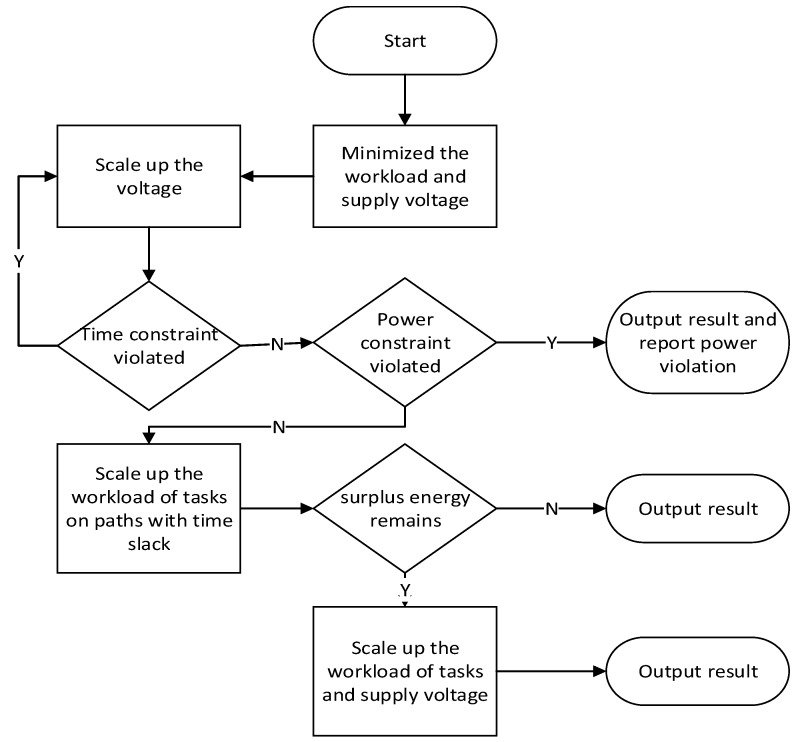
Flow chart of the configuration optimization algorithm.

A simple greedy algorithm is used to give an approximate optimal solution with an acceptable complexity for lightweight platforms. The basic thought of our algorithm is trading power for speed. Its principle is to satisfy the timing constraint first, and then scale up the workload and supply voltage to exploit the power and timing slacks. The configuration optimization can be expressed as the following steps as is shown in Figure 2:

1. Start from the configuration of the minimum workload and the lowest supply voltage, and then check the timing slacks. Increase the PE’s voltages and frequencies when execute the tasks on the paths having timing violations to meet the time constraint. A steepest drop algorithm is applied. It repeats scaling up the voltage and frequency of the task with the highest ratio of the performance improvement to the power increment and then updating the time slacks until all deadlines are met. For the mutual exclusive tasks, the paths are handled separately.
2. Check the power budget, if the energy slack is sufficient, go to Step 3, otherwise finish the optimization and output the result. Should there be a violation of power budget, this will be reported to the power budget planning module, which will take measures such as down regulating the power budget of the incoming time windows to compensate the energy deficit. For the conditional tasks, the product of their energy consumption and execution probability is taken as their energy for estimation instead.
3. Scale up the workload of the tasks on the paths with positive timing slack. This procedure starts from the path with the minimum timing slack and is repeated until no positive timing slack left on any path. During the workload scaling, the tasks on a path are scaled along the direction of edges. A task will be scaled after all the precedence tasks on this path have been scaled. In addition, the power budget will be updated after each task scaling, so that the scaling of the later tasks will not cause violation of the former scaled tasks. At that time, if no surplus energy budget remains, the optimization will finish, otherwise, go to Step 4.
4. Increase the workload of the tasks together with the voltage, ensuring that each task’s execution time is unchanged until all surplus energy is utilized. In this step, the tasks with higher reward/energy ratios are scaled first. If either the voltages of all PEs or the workloads of all tasks already reach their upper bounds, finish the optimization and output the result. The surplus energy will be stored for later use.

The flow chart for greedy algorithm is as shown Figure 2. The time complexity of the above algorithm is O(N^2^), where N is the number of the tasks.

## 6. Test Results

**Figure 3:**
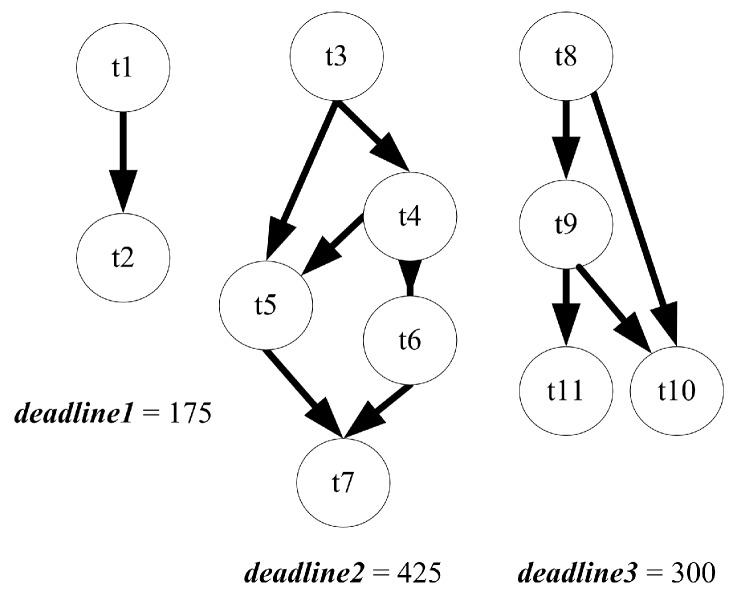
Example: a three programs application.

Three random TFGs, as Figure 3, are generated by TGFF [33] as a test case to demonstrate the procedure of the proposed solution. Each of them represents a program of an application on a system. The system hardware platform is a heterogeneous multi-core SoC with three PEs. PE1 and PE2 are general processors while PE3 is a specific accelerator which can only execute task t1 and t4. The parameters in term of performance and power consumption of each PE and tasks are set arbitrarily based on reasonability. Each program has its own deadline, which is shown under its TFG.

**Figure 4:**
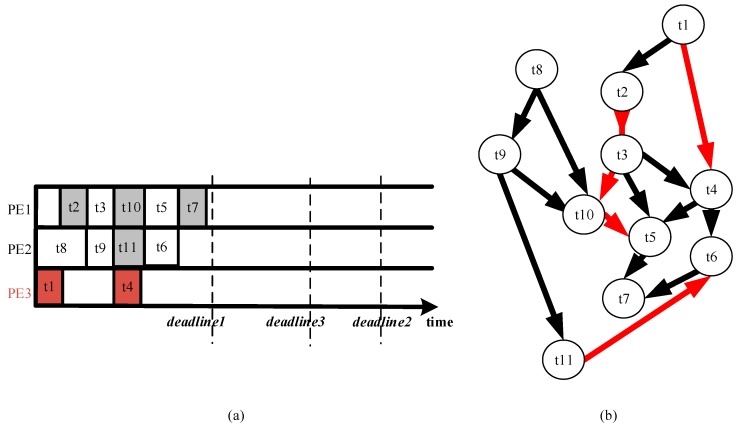
Offline task scheduling result. Each block represents a task scheduled, the width of which represents the execution time. (**a**) Scheduled scheme; (**b**) Result TFG with resource dependency.

We have used the proposed scheduling algorithm to schedule them into the platform. The obtained scheduling result is depicted in Figure 4. The execution PE and duration of the tasks can be learned from the Figure 4a, where each block represents a task scheduled. The height of the blocks represents the power consumption (also reflects the supply voltage) and the width represents the execution time. The result TFG which contains the resource dependency can be extracted from the schedule scheme and is shown in Figure 4b. In this new TFG, there are the nodes (tasks) and the data dependency edge of all input TFGs, which are colored black, in addition to some new edges colored red that reflect the resource sharing. For example, in the input TFGs, t1 and t4 belong to two independent programs, however, there is a new edge between them in the result TFG because they are assigned to the same PE.

**Figure 5:**
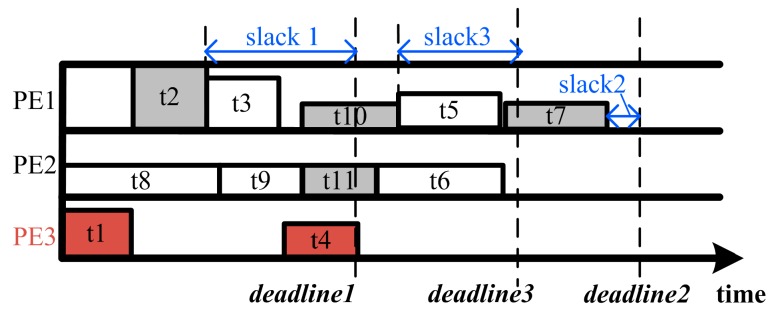
Configuration optimization result (Each block represents a task scheduled, the height of which represents the power consumption and the width represents the execution time).

When the result TFG is entered into the proposed on-line configuration optimization program, an optimized scheme under a given power budget can be obtained. Figure 5 is the result with the optimized configuration under a power budget. It can be seen that the tasks’ execution times are changed but their execution units and orders are unchanged, and that all deadlines are met in the final result. Regarding the system rewards, the reward of the initial task scheduling result of this example is 401 while that of the configuration optimized result is 481. The performance is improved.

**Figure 6:**
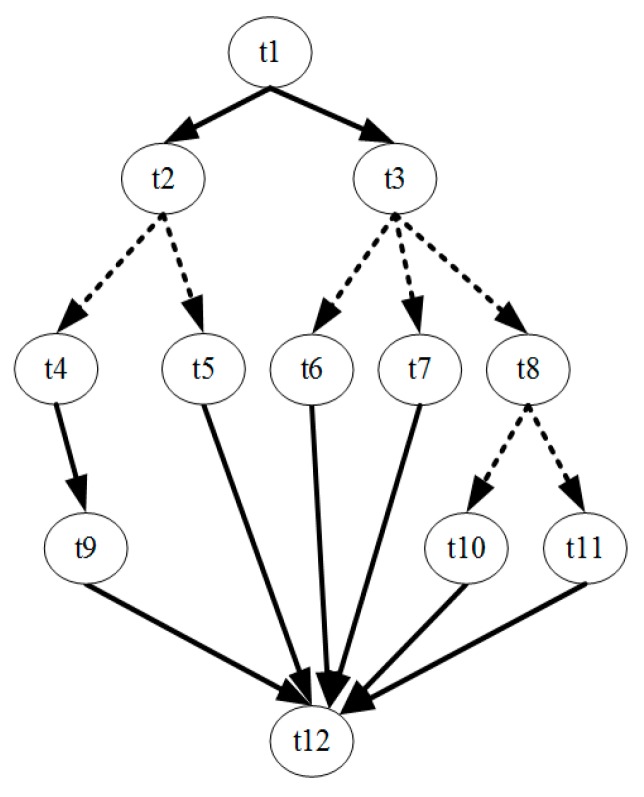
TFG of the test case.

**Figure 7:**
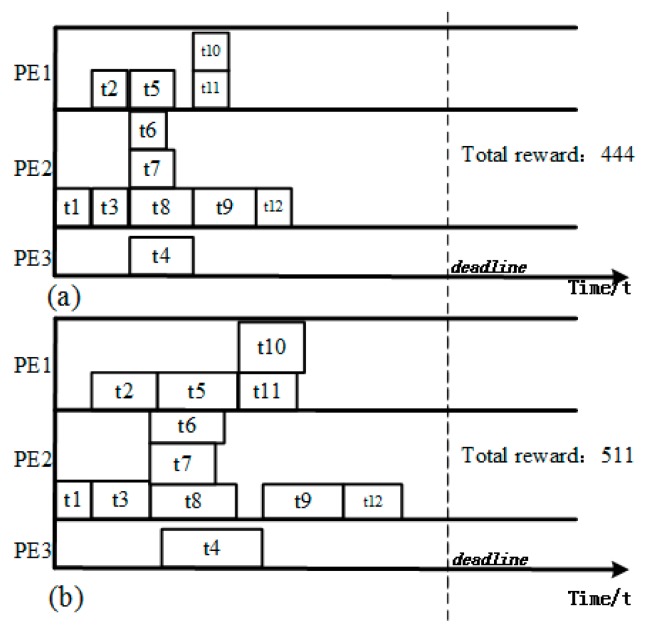
(**a**) Task scheduling result (without configuration optimization); (**b**) Configuration optimized result.

A second test that uses a random conditional branching TFG generated by TGFF has been performed to validate our algorithms. The TFG is as Figure 6 and the platform is still a 3-PE SoC, where PE1 and PE2 are general processors while PE3 is an accelerator for t4. The result of the task scheduling and configuration optimization for the TFG is shown in Figure 7. As is shown in the figure, the proposed method can give an optimized assignment and configuration for the tasks. The reward of the configuration optimized is better than that of the configuration without voltage and workload optimization. This result also illustrates that the proposed solution can handle branching cases correctly.

**Figure 8:**
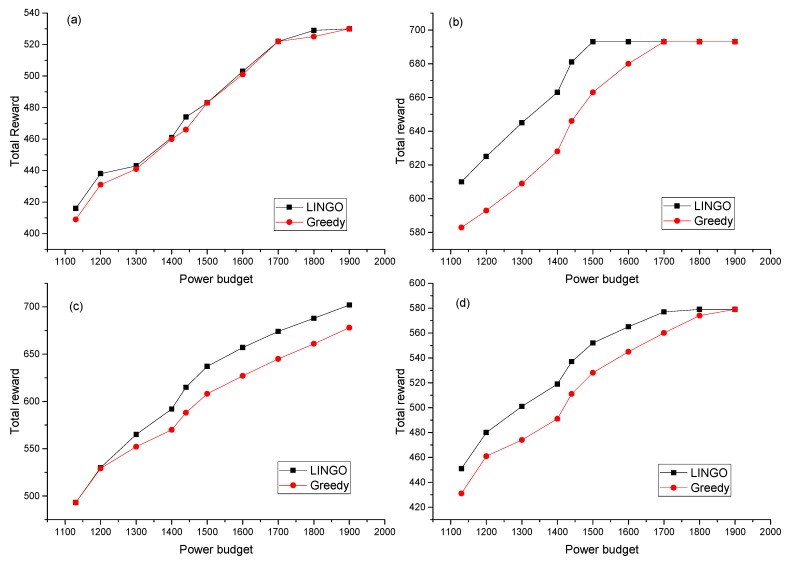
Comparison of results of greedy algorithm and LINGO. (**a**) Results of TFG 1 (19 tasks); (**b**) Results of TFG2 (18 tasks); (**c**) Results of TFG3 (18 tasks); (**d**) Results of TFG4 (17 tasks).

In order to evaluate the quality of the proposed greedy algorithm, a set of TFGs including 4 different random TFGs is generated as well, and then scheduled to the same heterogeneous SoC with the proposed scheduling algorithm. The total number of the tasks is set to be around 20 tasks, for considerations that this is sufficient for applications on our target light-weight platforms and that the resource limitation of such platforms. The scheduled result is then optimized by the proposed greedy algorithm under different power budgets. The algorithms proposed in previous works are not completely consistent with the application situation in this paper, the design considerations or the goals are different, thus the performance of the proposed algorithm is only compared with that of the classic solving software, LINGO. The test cases are optimized with the same constraints as a non-linear problem using the LINGO as reference. The result given by LINGO is regarded as the optimal result. The total system rewards of both solutions for one of the TFGs are compared in Figure 8. The figure shows that the total reward increases with the power budget for both algorithms, and the results of the other TFGs also have the same relationship, which suggests that our algorithm can exploit the harvested energy. Moreover, the usage ratios of power budget of all test cases are all higher than 99.9% and no power budget is violated, which indicates that our algorithm can fully utilize the available power energy budget.

**Figure 9:**
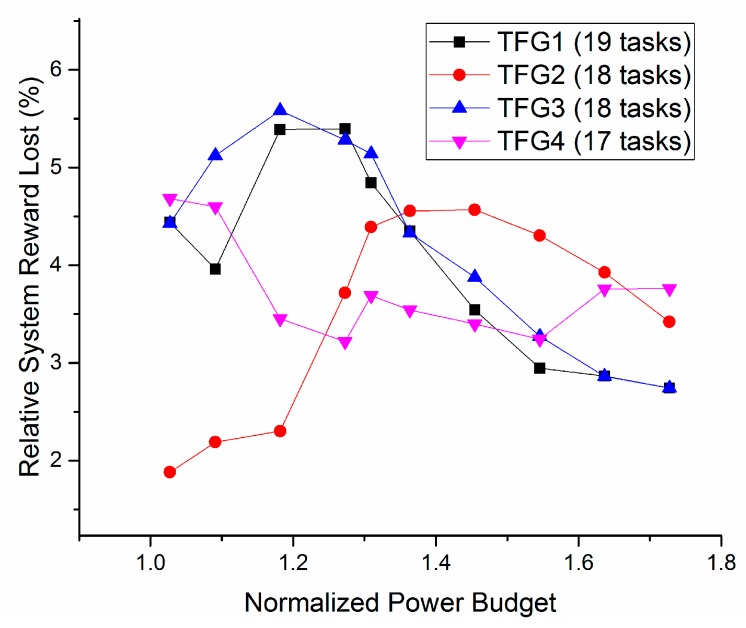
Relative system reward loss of our algorithm compared with the results given by LINGO, the power budget data are divided by the minimum power budget.

The resulting system rewards for the four cases of our algorithm and LINGO are compared in Figure 9. As shown, the total rewards of our algorithm are 4% in average worse than the results given by LINGO, and the worst case is 6%. This means the greedy algorithm only sacrifice a little performance. However, the LINGO solver uses a heuristic algorithm with the exponential time complexity, which costs minutes, even hours of computation time for tens of tasks, whereas our algorithm with a complexity of O(N^2^), where N is the number of the tasks, and costs only several milliseconds, running on the same PC platform. This advantage makes our algorithm capable of implementing on the light-weight platforms such as the WSN nodes.

The proposed configuration algorithm has been also compiled and implemented on an embedded MSP430 processor, using IAR System’s C/C++ compiler. The target device is a MSP430F6638, with a non-volatile memory of 256 KB and a RAM of 18 KB, the frequency of which is 20 MHz. The previous TFG with three programs and 19 tasks is used as test case here. Two more TFGs with four programs, 25 tasks and five programs, 45 tasks are also generated to evaluate the performance of the algorithm for larger task sets. The computational cost and memory footprint is shown in Table 1. For the medium size test cases with three programs and 19 tasks, it can finish the configuration optimization correctly in 357,842 clock cycles on the MCU platform, and for the larger test cases the cost is also acceptable, which proves that the algorithm can achieve real-time power management on light-weight platforms.

## 7. Conclusions

This paper presents a method of task scheduling and power management for the energy harvesting heterogeneous multi-core WSN node SoCs. It adjusts the workload of tasks jointly with the power mode of hardware to meet the varied real-time power budget. This approach enhanced the usage efficiency of the energy harvested. It resolves the complexity problem through offloading the task scheduling to the design phase and using a new greedy dynamic configuration optimization algorithm with O(N^2^) complexity. Additionally, two concepts, workload and system reward, are introduced as the unified measurements of the quantity of the works done and the quality of service respectively. The experimental results show that this solution can give the correct scheduling result and optimize the energy utilization efficiency, can handle branches in the WSN programs, and can achieve the real-time power management on light-weight platforms with 4% on average system reward lost.

## Figures and Tables

**Table 1:** Computational cost and memory footprint of the algorithm on MSP430.

Number of Tasks	Clock Cycle	CPU Time (ms)	Memory (Bytes)
19	357,842	17.9	15,072
25	728,543	36.42	28,874
45	3,387,457	169.37	137,872
